# Early pulmonary rehabilitation in ARDS patients: Effects on respiratory function and long-term outcomes: A retrospective study

**DOI:** 10.1097/MD.0000000000041023

**Published:** 2024-12-20

**Authors:** Li Da, Kai Zhang

**Affiliations:** a Department of Critical Care Medicine, The People’s Hospital of Wenjiang Chengdu, Chengdu, China; b Department of Orthopedics, The People’s Hospital of Wenjiang Chengdu, Chengdu, China.

**Keywords:** acute respiratory distress syndrome, intensive care unit, mechanical ventilation, pulmonary rehabilitation

## Abstract

This retrospective cohort study, conducted at People’s Hospital of Wenjiang Chengdu (January 2018 to January 2022), aimed to evaluate the effects of early pulmonary rehabilitation (PR) on respiratory function in acute respiratory distress syndrome patients after discontinuing invasive mechanical ventilation in the intensive care unit. Patients were divided into PR and control groups based on an offline training protocol. Outcome measures included short physical performance battery scores, respiratory muscle strength, and pulmonary function indicators at discharge and 3 months post-discharge, including peak expiratory flow, forced expiratory volume in 1 second/forced vital capacity, and vital capacity. Kaplan–Meier analysis assessed 6-month survival rates. Among the 240 patients, 120 received traditional training and 120 received early PR. Both groups had no significant differences in baseline characteristics. Short physical performance battery scores and respiratory muscle strength showed gradual improvements in both groups, with the PR group achieving significantly higher scores at 5 and 7 days (*P* < .05). At 3 months post-discharge, pulmonary function indicators such as peak expiratory flow, forced expiratory volume in 1 second/forced vital capacity, and vital capacity in the PR group were significantly improved compared to discharge and were higher than those in the control group (*P* < .05). The number of sputum aspirations in the PR group significantly decreased (*P* < .05), and intensive care unit stays after weaning were significantly shorter (*P* < .05). Kaplan–Meier survival analysis revealed that the cumulative survival rate at 6 months post-discharge was significantly higher in the PR group compared to the control group (hazard ratio = 0.660, 95% CI: 0.472–0.922, *P* = .014). These findings suggest that early PR significantly enhances respiratory function recovery, reduces complications, and improves survival outcomes in acute respiratory distress syndrome patients after invasive ventilation.

## 1. Introduction

Acute respiratory distress syndrome (ARDS) is a kind of acute lung injury characterized by alveolar-capillary damage following pulmonary assault, leading to a series of clinical syndromes.^[1]^ Amid the global spread of the COVID-19 by recent years, the proportion of ARDS cases caused by COVID-19 has increased.^[2]^ Clinically, ARDS patients typically present with severe respiratory distress, cyanosis, and tachycardia, posing a significant threat to their lives.^[3,4]^ As ARDS progresses, patients may develop symptoms including dyspnea and chest tightness. Even with high-concentration oxygen inhalation, clinical symptoms may persist, eventually leading to respiratory failure.^[5]^

Conventional clinical and nursing interventions, which primarily combine environmental and specialized care during treatment, are relatively simplistic and limited in their ability to protect and restore lung function.^[6,7]^ Clinical studies consistently show that mechanical ventilation severity and duration correlate with ARDS progression, leaving nearly half of discharged patients require rehospitalization within 6 months.^[8,9]^ These clinical findings underscore the urgent need for effective strategies to improve respiratory function and prognosis in ARDS patients. However, current clinical guidelines mainly focus on lung-protective ventilation and fluid resuscitation strategies,^[10]^ while the concept of pulmonary rehabilitation (PR) has yet to be firmly established in clinical practice. PR has been shown to significantly benefit patients with chronic respiratory diseases.^[5]^ PR is a thorough care for each individual to enhance physical and breach function by individualized care and related function training.^[11]^ In 2013, the American Thoracic Society first proposed the application of early PR training for ARDS patients.^[12]^ Additionally, PR has been shown to improve exercise capacity and quality of life in individuals with COPD and other chronic respiratory conditions.^[13]^ However, insufficient clinical evidence exists regarding the prognosis of ARDS patients undergoing invasive mechanical ventilation after extubation in the intensive care unit (ICU). Yuemei Feng et al^[14]^ also reported the benefits of PR training for the symptoms of ARDS. However, their study only included 50 patients, which represents a relatively small sample size, and it did not report on the complications experienced by the patients. These limitations are the significant drawbacks in the study. In this study, we completed a single-center study with 240 patients met the inclusion criteria, which could add the clinical evidence for the further intervention for patients with ARDS in ICU.

## 2. Methods

### 2.1. Study design and ethics approval

A retrospective cohort study was employed to select patients diagnosed with ARDS admitted to the East Campus ICU of People’s Hospital of Wenjiang Chengdu from January 2018 to January 2023. Ethics approval was obtained from the Ethics Committee of People’s Hospital of Wenjiang Chengdu (Approval No.: 2016-109).

### 2.2. Inclusion and exclusion criteria

Inclusion criteria: (1) age ≥ 18 years old; (2) time since weaning > 24 hours with stable hemodynamics; (3) no previous history of PR training; (4) patient’s family members must be able to cooperate and provide support and supervision during the postweaning rehabilitation training; (5) absence of significant cognitive impairment or severe neurological disorders.

Exclusion criteria: (1) re-intubation for mechanical ventilation postweaning; (2) hemodynamic instability, defined as dopamine > 10 mg/kg/min, adrenaline > 0.1 mg/kg/min, systolic blood pressure ≥ 170 mm Hg or ≤ 90 mm Hg (1 mm Hg ≈ 0.133 kPa), mean arterial pressure ≤ 65 mm Hg; (3) oxygenation index ≤ 100 mm Hg requiring extracorporeal membrane oxygenation or vasopressor therapy; (4) other severe complications such as acute stroke, active phase of seizures, active bleeding from any organ, or severe multi-organ dysfunction syndrome (MODS); (5) history of significant pulmonary fibrosis or COPD stage IV; (6) concurrent participation in other clinical trials that may interfere with the outcomes of this study; (7) inability to comprehend study procedures or provide informed consent due to language barriers or cognitive impairments.

### 2.3. Study methods

Patients were categorized into a control group and a PR group based on distinct weaning protocols. The control group underwent a conventional weaning training regimen, whereas the PR group received an early PR protocol developed from evidence-based research and initial clinical investigations conducted at our hospital. Aside from variations in weaning interventions, standard ICU practices governed the administration of other treatments and nursing care.

#### 2.3.1. PR group

Frequency and duration: the PR program was conducted 3 times per week for a duration of 6 weeks. Each session lasted approximately 45 to 60 minutes and was structured to include a warm-up, exercise, and cool-down phase.

Warm-up: the warm-up phase consisted of 5 to 10 minutes of low-intensity cycling or walking at a comfortable pace, performed on a stationary bicycle or in a designated walking area within the hospital.

Initial phase: patients began with passive limb movements performed at the bedside, focusing on major joint movements in all primary directions, with 20 repetitions per limb. Also perform additional back pats to help remove secretions.

Progression: patients who responded well to passive movements were encouraged to progress to active limb movements and deep breathing exercises. These exercises included moving from lying down to sitting upright to achieve static sitting balance, and pursed lip breathing, involving inhaling deeply through the nose and exhaling slowly through pursed lips, performed in 4 to 7 sets.

Advanced phase: patients who tolerated these activities advanced to task-specific functional exercises, such as abdominal breathing techniques performed lying flat with knees supported by a pillow, inhaling without chest movement to allow the abdomen to rise, and exhaling by compressing the abdomen to promote relaxation. Transfer training focused on moving from bed to chair, accompanied by instruction in effective coughing techniques to clear mucus.

Final phase: patients continued gait exercises and walking practice, incorporating pursed lip breathing, abdominal breathing, and effective coughing. Activities were progressively adjusted based on patient tolerance to ensure safe and effective progression.

Control group: in addition to pursed lip breathing, abdominal breathing, and effective coughing, the remaining activity protocols were identical to those of the observation group.

### 2.4. Outcome measures

#### 2.4.1. Short physical performance battery (SPPB) score

SPPB mainly consists of 3 components of assessment: balance testing, gait speed and pattern testing, and strength testing, each with specific scoring criteria. The total score ranges from 0 to 12 points. A high score indicates good physical function and mobility, while a low score reflects declined or limited physical function.^[15]^

Respiratory muscle strength (absolute values of maximum inspiratory pressure, maximum expiratory pressure, and sniff nasal inspiratory pressure). The assessment of respiratory muscle strength provides crucial information about the efficiency of respiratory muscle function, which can be used for diagnosis, monitoring disease progression, and evaluating the effectiveness of PR or mechanical ventilation weaning protocols.^[16]^

Pulmonary function indicators (respiratory rate, peak expiratory flow [PEF], forced expiratory volume in 1 second/forced vital capacity ratio [FEV1/FVC], vital capacity [VC]). Nursing staff conducted weekly telephone follow-ups with patients and their families. At the 3rd month post-discharge, patients were scheduled for respiratory outpatient clinic visits at our hospital.

### 2.5. Statistics methods

Data processing and analysis were performed using SPSS 23.0 statistical software. Data entry was conducted by 2 individuals. Continuous data conforming to normal distribution are expressed as mean ± standard deviation (x ± s). Between-group comparisons were analyzed using independent samples *t* test, and within-group comparisons were analyzed using paired *t* test. Categorical data are presented as frequencies (rates or proportions), and between-group comparisons were assessed using chi-square test. Kaplan–Meier survival curve analysis was used to assess the 6-month survival of ARDS patients weaned from invasive mechanical ventilation. Statistical significance was set at α = 0.05, with *P* < .05 indicating statistical significance.

## 3. Results

### 3.1. Characteristic of included patients

A total of 240 patients were ultimately included, with 120 undergoing traditional weaning training and 120 undergoing early PR training. There were no statistically significant differences between the 2 groups in terms of gender, age, APACHE II score upon admission, admission oxygenation index, ARDS etiology, duration of invasive mechanical ventilation, or mode of invasive mechanical ventilation (all *P* > .05; see Table 1).

**Table 1:** Comparison of characteristic between PR group and control group.

Variable	Total (n = 240)	C (n = 120)	PR (n = 120)	Statistic	*P*
Age, mean ± SD	48.13 ± 18.09	47.60 ± 17.71	48.66 ± 18.52	t = -0.450	.653
Gender, n (%)				χ² = 2.401	.121
Female	122 (50.83)	67 (55.83)	55 (45.83)		
Male	118 (49.17)	53 (44.17)	65 (54.17)		
APACHE II, mean ± SD	20.17 ± 8.89	19.81 ± 8.79	20.53 ± 9.01	t = -0.622	.534
PAO_2_, mean ± SD	223.52 ± 102.24	226.42 ± 104.51	220.63 ± 100.27	t = 0.438	.662
ARDS etiology, n (%)				χ² = 3.182	.528
Viral pneumonia	129 (53.75)	68 (56.67)	61 (50.83)		
Bacterial pneumonia	47 (19.58)	21 (17.50)	26 (21.67)		
Pyemia	34 (14.17)	15 (12.50)	19 (15.83)		
Atypical pathogenic bacterial pneumonia	10 (4.17)	7 (5.83)	3 (2.50)		
Other	20 (8.33)	9 (7.50)	11 (9.17)		
Ventilation mode, n (%)				χ² = 0.241	.886
PC	139 (57.92)	71 (59.17)	68 (56.67)		
SIMV	51 (21.25)	24 (20.00)	27 (22.50)		
SIMV + PC	50 (20.83)	25 (20.83)	25 (20.83)		
Ventilation time, mean ± SD	5.90 ± 0.99	5.85 ± 0.96	5.95 ± 1.03	t = -0.784	.434

ARDS = acute respiratory distress syndrome, C = control group, PC = pressure control, PR = pulmonary rehabilitation, SIMV = synchronized intermittent mandatory ventilation.

### 3.2. SPPB questionnaire scores

SPPB questionnaire scores (Table 2): no significant difference was found between the 2 groups for SPPB scores at 3 days (*P* > .05), but at 5 days and 7 days, PR group was superior to control group (8.45 ± 1.05 vs 9.58 ± 1.25; 9.54 ± 1.18 vs 10.48 ± 1.11, all *P* < .05). And both groups showed significantly increased SPPB scores (all *P* < .05).

**Table 2:** Comparison of SPPB questionnaire scores and respiratory muscle strength between PR group and control group.

Variable	Total (n = 240)	C (n = 120)	PR (n = 120)	Statistic	*P*
SPPB at 3 days, mean ± SD	7.71 ± 0.97	7.82 ± 1.06	7.60 ± 0.86	t = 1.823	.070
SPPB at 5 days, mean ± SD	9.01 ± 1.28	8.45 ± 1.05	9.58 ± 1.25	t = -7.602	<.001*
SPPB at 7 days, mean ± SD	10.01 ± 1.23	9.54 ± 1.18	10.48 ± 1.11	t = -6.386	<.001*
Statistic		F = 78.321	F = 209.588		
*P*		<.001*	<.001*		
Respiratory muscle strength at 3 days, mean ± SD	126.95 ± 25.70	129.48 ± 23.88	124.42 ± 27.26	t = 1.531	.127
Respiratory muscle strength at 5 days, mean ± SD	142.69 ± 26.31	137.30 ± 23.52	148.08 ± 27.91	t = -3.236	.001*
Respiratory muscle strength at 7 days, mean ± SD	151.20 ± 28.61	141.82 ± 24.87	160.58 ± 29.11	t = -5.368	<.001*
Statistic		F = 7.713	F = 51.244		
*P*		<.001*	<.001*		

PR = pulmonary rehabilitation, SPPB = short physical performance battery.

* *P* < .05, statistical significance.

### 3.3. Respiratory muscle strength

Respiratory muscle strength (Table 2): no significant difference was found between the 2 groups for respiratory muscle strength at 3 days (*P* > .05), but at 5 days and 7 days, PR group was superior to control group (137.30 ± 23.52 vs 148.08 ± 27.91; 141.82 ± 24.87 vs 160.58 ± 29.11, all *P* < .05). And both groups showed significantly increased respiratory muscle strength (all *P* < .05).

### 3.4. In-hospital outcomes

In-hospital outcomes (Table 3): following invasive mechanical ventilation weaning, patients in the PR group exhibited significantly lower frequencies of sputum aspirations (22.21 ± 1.77 vs 27.77 ± 1.84, *P* < .05), shorter ICU length of stay postweaning (6.90 ± 1.07 vs 8.52 ± 1.88, *P* < .05). Moreover, PR group had higher success rate of postweaning, lower complication rate, and lower rehospitalization rates within 6 months post-discharge compared to the control group (all *P* < .05).

**Table 3:** Comparison in-hospital outcomes between PR group and control group.

Variable	Total (n = 240)	C (n = 120)	PR (n = 120)	Statistic	*P*
Frequencies of sputum aspirations, mean ± SD	24.99 ± 3.32	27.77 ± 1.84	22.21 ± 1.77	t = 23.860	<.001*
Length of stay postweaning, mean ± SD	7.71 ± 1.73	8.52 ± 1.88	6.90 ± 1.07	t = 8.182	<.001*
Complications, n (%)	20 (8.33)	15 (12.50)	5 (4.17)	χ² = 5.455	.020*
Deep venous thrombosis	3	2	1	–	–
Urinary tract infection	3	3	0	–	–
Pressure ulcers	4	3	1	–	–
Others	10	7	3	–	–
Success rate of postweaning	207 (86.25)	98 (81.67)	109 (90.83)	χ² = 4.251	.039*
Rehospitalization rates	19 (7.92)	14 (11.67)	5 (4.17)	χ² = 4.630	.031*

PR = pulmonary rehabilitation.

* *P* < .05, statistical significance.

### 3.5. Pulmonary function indicators

Pulmonary function indicators (Table 4): upon discharge, there were no statistically significant differences in PEF, FEV1/FVC, and VC between the 2 groups (all *P* > .05), but PR group had lower respiratory rate compared to control group (*P* < .05). Moreover, at 3 months post-discharge, both groups showed significantly increased respiratory rate, PEF, FEV1/FVC, and VC compared to discharge values (all *P* < .05). Furthermore, the PR group exhibited significantly lower respiratory rate and higher PEF, FEV1/FVC, and VC compared to the control group at the 3-month follow-up (all *P* < .05).

**Table 4:** Comparison of pulmonary function indicators between PR group and control group.

Variable	Total (n = 240)	C (n = 120)	PR (n = 120)	Statistic	*P*
Respiratory rate at discharge, mean ± SD	22.24 ± 2.80	23.38 ± 2.17	21.09 ± 2.90	t = 6.923	<.001*
Respiratory rate at last follow-up, mean ± SD	19.16 ± 2.95	19.94 ± 2.61	18.37 ± 3.06	t = 4.255	<.001*
** **Statistic		t = 10.814	t = 6.535		
** ** *P*		<.001*	<.001*		
PEF at discharge, mean ± SD	302.27 ± 79.18	292.57 ± 74.12	311.97 ± 83.10	t = -1.909	.057
PEF at last follow-up, mean ± SD	398.34 ± 91.60	377.33 ± 78.94	419.35 ± 98.63	t = -3.644	<.001*
Statistic		t = -7.600	t = -9.208		
*P*		<.001**	<.001		
FEV1/FVC at discharge, mean ± SD	0.69 ± 0.10	0.68 ± 0.08	0.70 ± 0.11	t = -1.272	.205
FEV1/FVC at last follow-up, mean ± SD	0.83 ± 0.08	0.80 ± 0.07	0.87 ± 0.07	t = -8.292	<.001*
** **Statistic		t = -10.941	t = -14.962		
** ** *P*		<.001*	<.001*		
VC at discharge, mean ± SD	2.07 ± 0.34	2.04 ± 0.28	2.10 ± 0.40	t = -1.322	.188
VC at last follow-up, mean ± SD	2.92 ± 0.75	2.65 ± 0.59	3.19 ± 0.80	t = -6.017	<.001*
PEF statistic		t = -10.464	t = -13.274		
*P*		<.001*	<.001*		

FEV1/FVC = forced expiratory volume in 1 second/forced vital capacity ratio, PEF = peak expiratory flow, VC = vital capacity.

* *P* < .05, statistical significance.

### 3.6. Survival rate

**Figure 1.**
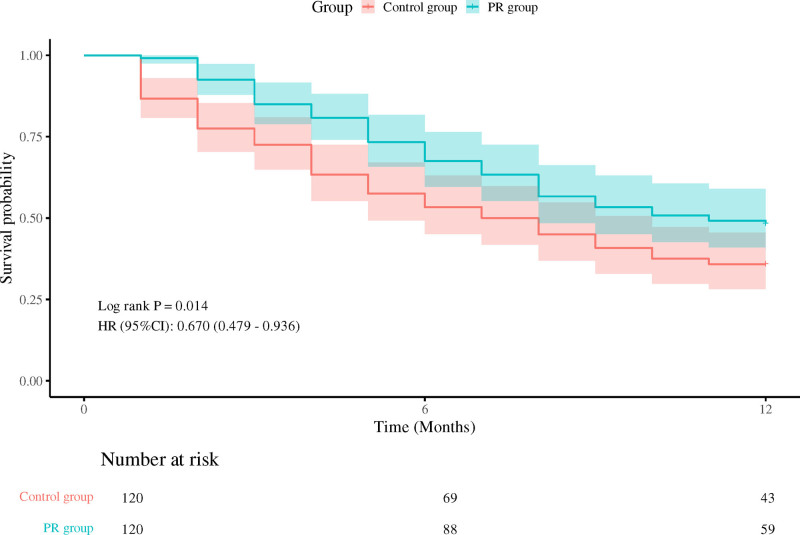
Survival at 6 months post-discharge.

Survival at 6 months post-discharge (Fig. 1): the Kaplan–Meier survival curve demonstrates that the cumulative survival rate of patients in the observation group is significantly higher than that of the control group (hazard ratio = 0.660, 95% CI: 0.472–0.922, *P* = .014).

## 4. Discussion

This study aims to evaluate the impact of early PR on respiratory function in ARDS patients in the ICU following the cessation of invasive mechanical ventilation. The results demonstrated that respiratory function indicators in the PR group were significantly superior to those in the control group, aligning with findings from relevant studies both domestically and internationally. A retrospective study has found that PR training significantly improves patients’ symptoms of ARDS and enhances their lung function.^[14]^

In this study, SPPB scores and respiratory muscle strength in the PR group were significantly higher than in the control group, consistent with Spruit et al’s findings in COPD patients, confirming PR’s effectiveness in enhancing respiratory muscle strength.^[12]^ In addition, early PR training significantly improved patients’ lung function parameters, including PEF, FEV1/FVC, and VC. This result is consistent with the finding by Schweickert et al in ICU patients, who showed that early exercise rehabilitation can improve overall lung function in patients.^[14,17]^ However, this study also found that the respiration rate in the PR group showed significant improvement compared to the control group (21.09 ± 2.90 vs 23.38 ± 2.17, *P* < .05). This could be attributed to PR training, which enhances the respiratory function of patients and increases their respiratory muscle strength.

The frequency of sputum aspiration and ICU stay in the PR group patients significantly decreased after weaning. The reduction in the frequency of suctioning suggests that early PR training may enhance patients’ spontaneous breathing ability and reduce their dependence on mechanical suctioning. This is consistent with the findings of Hao et al (2004) that PR can improve breathing function on patients with pulmonary diseases such as COVID-19.^[18]^ The reduction of hospitalization time further demonstrates the effectiveness of PR in promoting early recovery and reducing hospitalization time for patients, which is consistent with multiple research results at home and abroad.^[19]^

The Kaplan–Meier survival curve shows that the cumulative survival rate of PR group patients at 6 months after discharge is significantly higher than that of the control group, indicating that early PR training has a positive effect on improving long-term survival rate of patients. Seymour et al also showed in their study of ICU patients that early PR training can significantly improve patient survival rates.^[20]^ This result is consistent with our research, further demonstrating the importance of early PR training in improving the long-term prognosis of ARDS patients.

The findings of this study align with domestic research by Li et al, who reported that early PR significantly improved respiratory function and overall health in ICU patients.^[21]^ However, most domestic studies have focused on short-term effects, while this study provides longer-term follow-up data, showing the long-term effects of early PR training at 3 and 6 months after discharge. Additionally, our study further revealed differences in complications between the PR group and the control group, which included pressure ulcers, urinary tract infections, deep vein thrombosis of the lower extremities, and other related complications. Although there was no statistically significant difference in the incidence of each complication between the PR group and the control group (*P* > .05), the overall complication rate in the PR group was significantly lower than that in the control group (*P* < .05). It is evident that PR training can reduce the overall incidence of complications by improving patients’ lung function and increasing their time out of bed. Previous studies had not reported complications related to PR training in ARDS patients, but our study provides supplementary evidence to previous research, demonstrating a certain degree of novelty.^[14]^

Internationally, Rochwerg et al^[22]^ pointed out in a systematic review that PR has significant effects in improving respiratory function and reducing hospitalization time in ICU patients. Our findings are similar to conclusions of this review, further demonstrating the importance of PR in the rehabilitation of ARDS patients. In addition, some studies abroad have also pointed out that PR training can significantly improve patients’ mental health and quality of life. However, our study did not investigate these results, but it is also worth further attention in future studies.^[23]^

Early PR training improves respiratory function in ARDS patients through various mechanisms. Firstly, PR training strengthens respiratory muscles and enhances their endurance, improving patients’ autonomous breathing ability. Secondly, PR training promotes pulmonary function, reducing the occurrence of pulmonary inflammation and the cardiovascular risk.^[24–26]^ In addition, early PR training can enhance overall physical fitness, improve the overall health level of patients, and further promote the recovery of respiratory function.

The application of early PR training is of great significance in clinical practice. For ARDS patients, especially those who require long-term mechanical ventilation, early PR training can be an effective rehabilitation method to help patients recover spontaneous breathing function faster, reduce hospitalization time and medical costs, improve quality of life and long-term survival rate.^[27–29]^ Therefore, in ICU management, attention should be paid to the implementation of early PR training, and personalized rehabilitation plans should be developed to maximize patient recovery.

Although this study showed a positive impact of early PR training on ARDS patients, there are still some limitations. Firstly, as a retrospective cohort study, this research may have selection and information biases that could impact result validity. Secondly, the single-center design and relatively small sample size limit the generalizability, requiring validation through multicenter prospective studies with larger samples.^[30]^ Third, the follow-up is too short to provide information regarding the long effect of PR, and we plan to extend the follow-up period in future research to assess the lasting effects of early PR. In addition, this study failed to comprehensively evaluate the specific content and intensity of PR training on patients’ respiratory function. Future research should further explore the effects of different PR training programs to optimize rehabilitation strategies.^[31,32]^

## 5. Conclusion

Early PR significantly enhances respiratory function in ARDS patients after weaning from invasive mechanical ventilation. Continuous respiratory training post-discharge further improves function and reduces mortality. However, high-quality randomized controlled trials are required to validate these findings due to the study’s limitations.

## Author contributions

**Conceptualization:** Kai Zhang, Li Da.

**Data curation:** Kai Zhang, Li Da.

**Formal analysis:** Kai Zhang, Li Da.

**Funding acquisition:** Kai Zhang.

**Investigation:** Kai Zhang, Li Da.

**Methodology:** Kai Zhang, Li Da.

**Project administration:** Kai Zhang, Li Da.

**Resources:** Kai Zhang, Li Da.

**Software:** Kai Zhang, Li Da.

**Supervision:** Kai Zhang, Li Da.

**Validation:** Kai Zhang, Li Da.

**Visualization:** Kai Zhang, Li Da.

**Writing – original draft:** Kai Zhang, Li Da.

**Writing – review & editing:** Kai Zhang, Li Da.
